# Consciously processing balance leads to distorted perceptions of instability in older adults

**DOI:** 10.1007/s00415-020-10288-6

**Published:** 2020-11-03

**Authors:** Toby J. Ellmers, Elmar C. Kal, William R. Young

**Affiliations:** 1 School of Sport and Health Sciences, University of Exeter, Exeter, UK; 2 The College of Health, Medicine and Life Sciences, Brunel University London, Uxbridge, London, UB8 3PH UK; 3 Centre for Cognitive Neuroscience, Brunel University London, London, UK

**Keywords:** Fear of falling, Anxiety, Dizziness, Older adults, Conscious movement processing, Balance

## Abstract

**Background:**

Persistent dizziness without a clear cause is common in older adults. We explored whether an anxiety-driven preoccupation with consciously processing balance may underpin the distorted perceptions of unsteadiness that characterises ‘unexplained’ dizziness in older adults.

**Methods:**

We experimentally induced anxiety about losing one’s balance (through a postural threat manipulation) in a cohort of asymptomatic older adults and evaluated associated changes in perceived stability, conscious movement processing and postural control. These outcomes were also assessed when performing a distracting cognitive task designed to prevent anxiety-related conscious movement processing, in addition to during baseline conditions (ground level).

**Results:**

Despite a lack of increase in postural sway amplitude (*p* = 0.316), participants reported reductions in perceived stability during postural threat compared to baseline (*p* < 0.001). A multiple linear regression revealed that anxiety-related conscious movement processing independently predicted perceptions of instability during this condition (*p* = 0.006). These changes were accompanied by alterations in postural control previously associated with functional dizziness, namely high-frequency postural sway and disrupted interaction between open- and closed-loop postural control (*p*s < 0.014). While the distraction task successfully reduced conscious processing (*p* = 0.012), leading to greater perceived stability (*p* = 0.010), further increases in both postural sway frequency (*p* = 0.002) and dominance of closed-loop control (*p* = 0.029) were observed.

**Conclusion:**

These findings implicate the role of conscious movement processing in the formation of distorted perceptions of unsteadiness, suggesting that such perceptions may be modifiable by reducing an over-reliance on conscious processes to regulate balance.

## Introduction

Dizziness affects approximately 30% of those aged above 65 years [1], and is associated with self-reported functional disability [2, 3], poorer overall health [4], disrupted balance and increased falls in this population [1, 5]. Dizziness is a subjective complaint that, in older adults, is typically reported as a vague sensation of unsteadiness [1, 6]. Chronic dizziness is often attributable to specific neurological, cardiovascular or vestibular dysfunction. However, in at least 50% of cases in older adults, dizziness cannot be explained through traditional neurological or neuro-otological testing [6–8]. This does not necessarily mean that neurological or neuro-otological dysfunction is absent, but rather that symptoms of dizziness cannot be readily attributed to such dysfunction using currently available clinical tests. Without an attributable cause, treating the high prevalence of dizziness that occurs in this population represents a significant challenge.

In the case of Persistent Postural-Perceptual Dizziness (PPPD), a newly defined disorder of functional dizziness that mostly affects middle-aged adults, recent work suggests that attentional (and related neuro-cognitive) factors may underpin dizziness symptoms that do not present a clear neurological or neuro-otological basis [6, 9–13]. Specifically, it has been suggested that PPPD symptoms may be caused by an anxiety-related preoccupation with consciously processing balance [6, 9–13]. Such hypervigilance is purported to lead to greater awareness of (otherwise subconscious) balance sensations, eliciting a scaling ‘mismatch’ between perceived and actual postural movements. This mismatch is then experienced as a distorted sense of unsteadiness. Given that older adults frequently experience anxiety about falling [14], with such anxiety reliably linked to increased conscious balance processing [15, 16], similar neuro-cognitive factors might also underpin ‘unexplained’ dizziness in older adults. Indeed, recent research has shown that anxiety is associated with increased dizziness symptoms in this population [17, 18]. It remains unknown if this relationship is causal in nature, and, if so, what the specific underlying mechanisms are.

The present work aimed to scrutinise the relationship between fall-related anxiety, conscious processing of balance, and symptoms of dizziness in older adults. Specifically, we sought to experimentally induce attentional factors implicated in existing models of PPPD (anxiety-related conscious processing of balance [6, 9–13]) in a cohort of asymptomatic older adults and evaluate associated changes in perceived instability and postural control. An asymptomatic cohort was selected to isolate the effects of attentional factors on dizziness symptoms, independent from vestibular or neurological dysfunction. Thus, any observed distorted perceptions of instability (and/or postural control strategies associated with dizziness) following the experimental manipulation would provide strong support for the role of attentional factors in the presentation of these symptoms. We predicted that anxiety would lead to both a distorted sense of unsteadiness and maladaptive postural control strategies previously associated with dizziness: high-frequency postural sway and disrupted interaction between open- and closed-loop postural control (but no changes in overall sway amplitude) [12, 13]. We hypothesised that these outcomes would be a consequence of increased conscious processing of balance. We, therefore, also predicted that preventing participants from consciously processing their balance (through a distracting secondary cognitive task) would successfully reduce perceptions of instability (while having limited effect on *actual* postural stability) during experimental conditions of heightened anxiety.

## Methods

### Participants

Previous research has reported large effect sizes for comparable outcome variables when young adults performed a distracting secondary cognitive task during conditions of postural threat [19]. A power analysis subsequently revealed that 15 participants would be required to obtain 80% power when using multiple paired-samples *t* tests (alpha adjusted for multiple comparisons, using the Holm–Bonferroni Method [20]). Twenty-six community-dwelling older adults (aged > 60 years; males: 7/26; mean ± SD age: 74.23 ± 6.98) were recruited to participate in the primary research exploring perceptions of stability and postural responses between conditions of Baseline, Threat and Threat-Distraction (see Procedures section below for description of each condition). For our secondary (regression) analysis, a power calculation revealed that 42 participants would be required detect a significant improvement in *R*^2^ (of 0.25) when adding conscious movement processing and fear of falling to a linear regression model seeking to predict perceived stability (*α* = 0.05, *β* = 0.80; with 3 total predictors: postural sway (control variable), conscious movement processing and fear of falling). Therefore, for this analysis, the current dataset was combined with previously unpublished data from 18 community-dwelling older adults (aged > 60 years; males: 6/18; mean ± SD age: 73.44 ± 7.11) who participated in an earlier study that compared identical outcome variables during conditions of Baseline and Threat only (i.e. not Threat-Distraction). Note, these participants did not significantly differ from those that completed the full protocol on any assessed demographic variable (see Table 1 for list of variables; all *p *values for comparisons > 0.12), nor were there any significant between-group differences for any outcome during either Baseline (all *p *values > 0.19) or Threat (all *p *values > 0.13).

**Table 1:** Demographic data for both the primary (Baseline vs. Threat vs. Threat-Distraction) and secondary (regression) analysis

Mean (SD)^a^	Primary analysis (*n* = 26)	Secondary (regression) analysis (*n* = 44)
Age	74.23 (6.98)	73.91 (6.96)
Gender, males (%)	7/26 (26.92%)	13/44 (29.55%)
Height (cm)	164.38 (8.66)	164.82 (9.13)
Weight (kg)	71.62 (17.41)	70.70 (15.42)
Berg balance scale (0–56)	52.35 (3.09)	52.96 (2.68)
Timed up and go (s)	11.30 (3.27)	10.67 (2.81)
Montreal cognitive assessment (0–30)	26.54 (2.92)	26.61 (2.83)
Falls in previous year, no. of participants (%)	8/26 (30.77%)	14/44 (31.82%)
No. daily medications	2.56 (2.02)	2.61 (2.83)
Falls Efficacy Scale-International	21.96 (4.88)	21.93 (4.50)

^a^Unless stated otherwise, variables are reported as the mean (and standard deviation)

All participants were recruited from local community groups, and were free from any cardiovascular or musculoskeletal impairment that prohibited them from standing for > 2 min without support. Participants did not report a current diagnosis for any neurological or vestibular condition, nor did they report any recent (within the past 6 weeks) bouts of dizziness. Participants were excluded if they demonstrated major cognitive impairment (Montreal Cognitive Assessment (MoCA) score < 18/30 [21]), or if they were currently prescribed medication for anxiety. Demographic information for both cohorts is reported in Table 1. Institutional ethical approval was obtained from the local ethics committee and the research was carried out in accordance with the principles laid down by the Declaration of Helsinki. All participants provided written informed consent prior to participation.

### Procedures

**Fig. 1:**
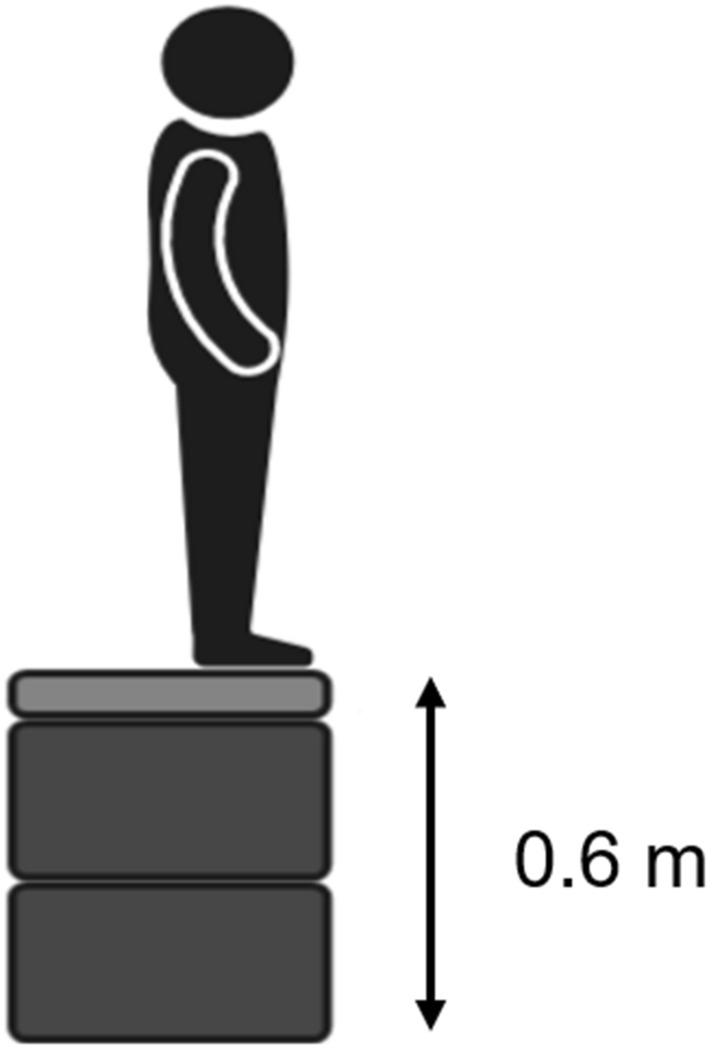
Schematic diagram of the postural threat manipulation used during both the Threat and Threat-Distraction conditions. Note, participants completed all trials without a safety harness

Participants completed narrow-stance (feet 10-cm apart) balance trials while standing on the edge of a force platform (Accusway, AMTI Inc., Watertown, MA, USA). Position of the feet was marked to ensure consistency between trials. Participants stood with their hands by their sides looking straight ahead at a cross affixed to the wall 3 m away. Participants completed a single 60-s trial under the following conditions: Baseline (ground level), Threat (inducing anxiety about falling by standing on the edge of a platform raised to 0.6 m, see Fig. 1) and Threat-Distraction. This final condition was identical to Threat, with the exception that participants also completed a distracting concurrent task while standing on the raised platform. This required participants to continuously verbalise the months of the year in the correct order, starting with January, for the duration of the 60-s trial. This task was chosen as it should substantially restrict participants’ opportunity to consciously process aspects of their balance performance—but at the same time the task presents a minimal cognitive demand and is, therefore, not expected to impair balance control due to increased task difficulty. Threat and Threat-Distraction trials were presented in a counterbalanced order, but these trials were completed prior to Baseline. Prior to participation, all participants first completed a 30-s practice trial at ground level. All trials were completed without a safety harness. Participants reached the raised platform via a series of wooden steps.

Immediately prior to each trial (i.e. while standing in position) participants rated how confident they were that they could maintain their balance and avoid a fall (0–100% confident) [22, 23]. After each trial, participants rated the level of fear of falling they experienced during the trial itself (0–100% fearful) [22, 23]. At this point, participants also completed a 4-item questionnaire that assessed the occurrence of conscious balance processing (e.g. “I am always trying to think about my balance when I am doing this task”; 1 = *strongly disagree*; 6 = *strongly agree*) [24]. Finally, given that dizziness in older adults is typically experienced as a distorted sense of instability [1, 6], participants also rated the subjective stability that they experienced during the preceding trial (0–100% stable) [23].

### Data analysis

Centre-of-pressure data from the force plate were sampled at 500 Hz. Data were low-pass (5 Hz) filtered offline with a bidirectional, second-order Butterworth filter. We first assessed the amplitude, frequency, and complexity of postural adjustments by calculating the root mean square (RMS) [22, 23, 25], mean power frequency (MPF; mean frequency in power spectrum after fast Fourier transformation) [22, 23, 25], and sample entropy (SampEn) of centre-of-pressure (COP) data [26, 27]. Height-induced postural threat has been shown to increase MPF due to concurrent reductions in low-frequency COP oscillations and increases in high-frequency COP oscillations (the latter of which is thought to reflect anxiety-related postural stiffening) [28]. Thus, average COP power within specific frequency ranges of 0–0.05 Hz (Freq_low_), 0.5–1.8 Hz (Freq_med_), and 1.8−5 Hz (Freq_high_) were also calculated [25]. SampEn is a measurement of movement complexity. Higher values reflect more complex and irregular postural adjustments, which is characteristic of more automatic (i.e. less consciously processed) postural control [29]. We optimised the parameter settings required for the SampEn calculation, resulting in the use of *m* = 3 and *r* = 0.01 [26]. Given that the postural threat (platform edge) was anterior to participants, all analyses were confined to anterior–posterior (AP) direction [22, 23, 25], and RMS and MPF were calculated with respect to the COP mean position [22, 25].

**Fig. 2:**
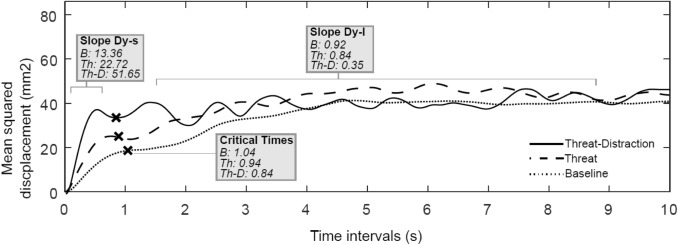
Representative stabilogram diffusion plots for a single participant during conditions of Baseline (‘*B*’), Threat (‘*Th*’) and Threat-Distraction (‘*Th-D*’). Note, the short- and long-term diffusion coefficients are indicated by the slopes Dy-s and Dy-l, respectively

To provide insight into open- and closed-loop control of posture, stabilogram diffusion analysis (SDA) was performed on COP displacement in the AP direction using the method described by Collins and De Luca [30, 31]. SDA plots mean squared COP displacements as a function of the time interval over which they occur. As illustrated in Fig. 2, SDA plots reveal two regions (short- and long-term diffusion) separated by a critical point where postural control is argued to move from predominantly open- to closed-loop control (i.e. the point at which sensory feedback is used to control posture) [30, 31]. During short-term intervals, COP exhibits persistent behaviour, tending to drift away from a relative equilibrium point. During longer term intervals, however, sensory feedback is used to return the COP to equilibrium (i.e. anti-persistent behaviour). The following variables were calculated during SDA: the critical time period (s) and displacement (mm^2^) at which closed-loop control begins to predominate in postural sway behaviour, in addition to short- and long-term diffusion coefficients (termed *D*_s_ and *D*_l_, respectively, and measured in mm^2^/s). To ensure that our SDA and SampEn outcomes were comparable to previous research that collected data at 100 Hz [26, 27, 30, 31], force plate data were down-sampled to 100 Hz for both SampEn and SDA analyses. Note, doing so affects only the values returned, not the overall pattern of results.

### Statistical analysis

Statistical analyses were conducted in three steps. First, separate paired-samples *t* tests were used to compare differences between Baseline and Threat for all variables. Second, separate paired-samples *t* tests were used to compare differences between Threat and Threat-Distraction. In both steps, Wilcoxon tests were used for comparisons involving non-normally distributed data. Effect sizes are reported as Cohen’s *d* for *t* tests, and *r* = *Z*/√ for Wilcoxon tests. Two-tailed tests were used for all comparisons. Alpha was corrected using the Holm–Bonferroni Method to account for multiple comparisons [20], and adjusted *p *values are reported. Finally, a linear regression analysis was performed to isolate the influence of conscious movement processing on perceptions of instability during conditions of Threat. Self-reported stability during Threat served as the dependent variable. To control for actual postural sway, RMS (during Threat) was entered in the first step as a control variable, with conscious movement processing and fear of falling (both also during Threat) added in the second step. It was not possible to add balance confidence into the model due to multicollinearity issues. All effects are reported in their unstandardised form. The assumptions of homoscedasticity (plot of standardised predicted values vs. residuals), error-independence (Durbin-Watson values = 2.16), lack of multicollinearity (*r*s = 0.010–0.475, variance inflation factors and tolerances < 1.52), and normal distribution of errors (non-significant Kolmogorov–Smirnov) were met.

### Data availability

All analysed data are available via an Open Science Framework repository (https://osf.io/nvrky/?view_only=bcd27ccfe99c47feaff215173a76d2fc).

## Results

Please see Table 2 for mean values (and standard deviation) for all assessed variables.

**Table 2:** Mean, standard deviation (SD) and *p* values for comparisons between Baseline (‘B’), Threat (‘Th’) and Threat-Distraction (‘Th-D’) trials for all outcome variables

	Baseline		Threat		Threat-Distraction		B. vs. Th	Th. vs. Th-D
	Mean	SD	Mean	SD	Mean	SD	*p*	*p*
Fear of falling (%)	4.63	10.82	21.92	23.28	21.35	24.27	**0.002**	1.00
Balance confidence (%)	95.39	10.58	75.19	22.43	80.00	23.66	** < 0.001**	0.115
Conscious movement processing	11.50	4.66	13.19	4.61	10.62	5.00	**0.019**	**0.012**
Perceived stability (%)	90.58	11.34	70.19	24.39	78.08	22.94	** < 0.001**	**0.010**
RMS (COP amplitude, mm)	5.24	2.38	4.80	1.26	4.43	1.20	0.316	0.060
MPF (COP frequency, Hz)	0.26	0.13	0.35	0.16	0.43	0.19	**0.002**	**0.002**
Frequency_low_ (mm^2^/bin)	191.87	236.92	112.58	137.80	88.96	99.20	**0.026**	0.790
Frequency_medium_ (mm^2^/bin)	2.19	1.59	3.23	2.63	3.92	3.33	**0.002**	0.209
Frequency_high_ (mm^2^/bin)	0.07	0.06	0.13	0.13	0.20	0.21	**0.001**	** < 0.001**
Sample entropy (movement complexity)	0.35	0.19	0.42	0.15	0.50	0.11	**0.007**	** < 0.001**
Critical time period (s)	1.21	0.41	1.02	0.39	0.93	0.19	**0.014**	**0.029**
Critical displacement (mm^2^)	38.27	47.86	27.11	17.30	28.35	19.58	1.00	1.00
Short-term diffusion (*D-y*_s,_ mm^2^/s)	17.89	13.36	22.57	15.79	26.93	20.50	**0.012**	0.191
Long-term diffusion (*D-y*_l,_ mm^2^/s)	1.37	2.20	0.95	1.51	0.52	0.78	0.165	0.195

Statistically significant differences (α < 0.05) are identified in bold

All *p *values are adjusted for multiple comparisons, using the Holm–Bonferroni Method

## Baseline vs. Threat comparisons

**Fig. 3:**
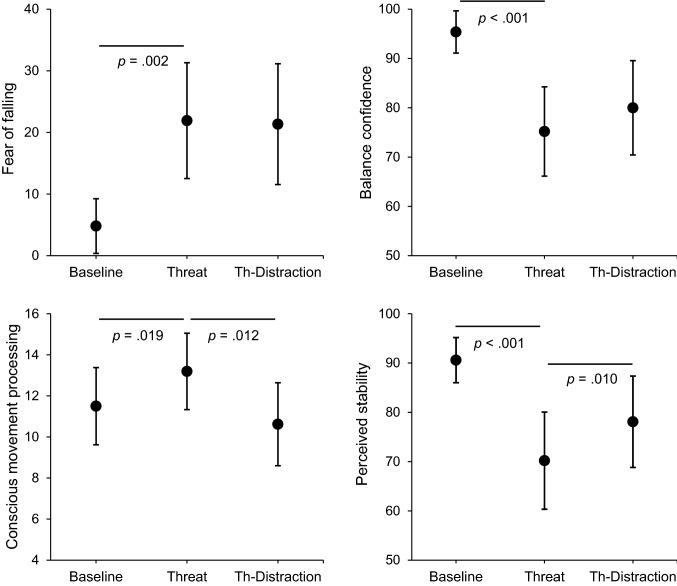
Mean (and 95% confidence intervals) for all self-reported outcome variables. The threat manipulation resulted in both increases in fear of falling (top left) and reductions in balance confidence (top right). These changes were accompanied by increases in conscious movement processing (bottom left) and reductions in perceived stability (bottom right). However, as illustrated in the bottom panels, performing the distracting cognitive task during conditions of postural threat (Threat-Distraction condition) resulted in increased perceptions of stability, in conjunction with reductions in conscious movement processing; but no significant change in either fear of falling or balance confidence. Note, all *p *values are adjusted for multiple comparisons using the Holm–Bonferroni Method

Participants reported greater perceived unsteadiness during Threat (*Z* = − 4.10, *p* < 0.001, *r* = 0.80). This was accompanied by significant increases in both conscious movement processing (*Z* = − 2.34, *p* = 0.019, *r* = 0.46) and fear of falling (*Z* = − 3.42, *p* = 0.002, *r* = 0.67), as well as significant reductions in balance confidence (*Z* = − 3.94, *p* < 0.001, *r* = 0.77) (Fig. 3).

**Fig. 4:**
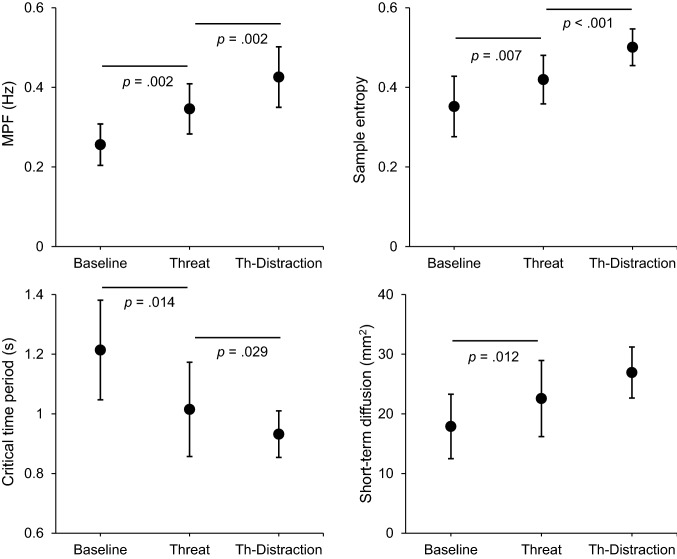
Mean (and 95% confidence intervals) for key postural control outcome variables. The threat manipulation resulted in increases in MPF (COP frequency; top left), sample entropy (movement complexity; top right) and short-term diffusion (bottom right), in conjunction with reductions in the critical time period (bottom left). These patterns of results appeared to be further maximised when performing the distracting cognitive task during conditions of postural threat (Threat-Distraction condition), with further increases in MPF and sample entropy, and further reductions in the critical time period, observed during the Threat-Distraction condition. Note, all *p *values are adjusted for multiple comparisons using the Holm–Bonferroni Method

Despite a lack of significant change in postural sway between conditions (RMS: *Z* = − 1.00, *p* = 0.316, *r* = 0.20), postural threat resulted in significant increases in MPF (*t*(25) = − 3.98, *p* = 0.002, *d* = 0.62). This was due to significant reductions in Freq_low_ (*Z* = − 2.48, *p* = 0.026, *r* = 0.49), and concomitant significant increases in both Freq_med_ (*Z* = − 3.21, *p* = 0.002, *r* = 0.63) and Freq_high_ (*Z* = − 3.24, *p* = 0.001, *r* = 0.64). In addition, SampEn significantly increased during Threat (*t*(25) = − 2.92, *p* = 0.007, *d* = 0.39) (Fig. 4).

With respect to the SDA, a significant shortening of the critical time period was observed during Threat (*Z* = − 2.69, *p* = 0.014, *r* = 0.53), which occurred in conjunction with significant increases in short-term diffusion coefficients (*Z* = − 2.73, *p* = 0.012, *r* = 0.54). There was a lack of significant between-condition change in either critical displacement (*Z* = − 0.34, *p* = 1.00, *r* = 0.07) or long-term diffusion coefficients (*Z* = − 1.73, *p* = 0.165, *r* = 0.34) (Fig. 4).

### Threat vs. Threat-Distraction comparisons

Performing the distraction task resulted in significantly greater subjective postural stability (*Z* = − 2.58, *p* = 0.010, *r* = 0.51), and significant reductions in conscious movement processing (*Z* = − 2.75, *p* = 0.012, *r* = 0.54). There was a lack of significant change in either fear of falling (*Z* = 0.00, *p* = 1.00, *r* = 0.00) or balance confidence (*Z* = − 1.58, *p* = 0.115, *r* = 0.31)  (Fig. 3).

There was a lack of significant change in RMS during Threat-Distraction (*t*(25) = 2.30, *p* = 0.060, *d* = 0.30). However, the distraction task led to significant increases in MPF (*t*(25) = − 3.54, *p* = 0.002, *d* = 0.47), an effect that was underpinned by significant increases in Freq_high_ (*Z* = − 3.49, *p* < 0.001, *r* = 0.69), but not by changes in Freq_low_ (*Z* = − 0.27, *p* = 0.790, *r* = 0.05) or Freq_med_ (*Z* = − 1.26, *p* = 0.209, *r* = 0.25). SampEn also increased significantly from Threat to Threat-Distraction (*t*(25) = − 4.57, *p* < 0.001, *d* = 0.61) (Fig. 4).

With respect to the SDA, the critical time period was significantly shorter during Threat-Distraction (*Z* = − 2.19, *p* = 0.029, *r* = 0.43). No significant changes were found for critical displacement (*Z* =− 0.22, *p* = 1.00, *r* = 0.04), short-term diffusion coefficient (*Z* = − 1.31, *p* = 0.191, *r* = 0.26), or long-term diffusion coefficient (*Z* = − 1.30, *p* = 0.195, *r* = 0.26) (Fig. 4).

### Regression analyses

While actual postural sway (RMS) did not predict perceived stability during Threat (*F* = 0.61, *R*^2^ = 0.014, *p* = 0.438), adding conscious movement processing and fear of falling significantly improved model fit, accounting for an additional 32.6% variance during Threat (*F* = 6.88, *R*^2^ = 0.340, *p* = 0.001; Table 3). However, only conscious movement processing (*B* = − 2.241, *p* = 0.006)—and not fear of falling (*B* = − 0.195, *p* = 0.153)—independently predicted perceptions of stability.

**Table 3:** Hierarchical regression model with conscious movement processing and fear of falling as a predictor of perceived stability, when controlling for postural sway amplitude (RMS)

Perceived stability	*B* (SE)	[95% CI]	*p*	*R*^2^	*R*^2^ change
Step 1				0.014 (*p* = 0.438)	
Constant	61.774 (12.539)	[36.470, 87.078]	** < 0.001**		
Postural sway amplitude (RMS)	1.938 (2.473)	[− 3.052, 6.928]	0.438		
Step 2				0.340 (***p = 0.001***)	0.326
Constant	98.460 (14.208)	[69.745, 127.175]	** < 0.001**		
Postural sway amplitude (RMS)	1.598 (2.080)	[− 2.606, 5.802]	0.447		
Conscious movement processing	− 2.241 (0.770)	[− 3.797, − 0.685]	**0.006**		
Fear of falling	− 0.195 (0.134)	[− 0.465, 0.075]	0.153		

Statistically significant differences (α < 0.05) are identified in bold

*RMS* Root mean square

## Discussion

The present work describes how healthy, asymptomatic older adults will experience a reduction in perceived stability during an anxiety-inducing postural threat manipulation. As these changes were not accompanied by increased postural sway amplitude, these perceptions can be argued to reflect a distorted sense of unsteadiness—a key symptom of functional dizziness disorders, such as PPPD, as well as idiopathic dizziness in older adults. While previous research has described an association in older adults between anxiety and both increased dizziness symptoms [17, 18] and perceived instability [32], the present work implies that this relationship may be causal in nature. Further, our results strongly indicate that such anxiety-related perceptions of instability are driven—at least in part—by heightened conscious processing of balance [6, 9–13]. During Threat, perceptions of instability occurred in conjunction with greater self-reported conscious processing of balance. While *actual* postural sway failed to predict perceptions of stability during Threat (accounting for around 1% of variance), conscious movement processing significantly—and independently—predicted perceived stability (see Table 3). Furthermore, anxious participants who were prevented from consciously processing their balance through the performance of a distracting concurrent task (Threat-Distraction condition) reported feeling significantly more stable compared to Threat (in the absence of any significant changes in postural sway amplitude, fear of falling or balance confidence). These findings strongly implicate the role of conscious movement processing in the formation of distorted perceptions of unsteadiness, suggesting that such perceptions may be modifiable through cognitive therapies that reduce the reliance on conscious processing to regulate balance.

In older adults, dizziness is typically reported as a vague sensation of unsteadiness [1, 6]. Despite the present cohort reporting an absence of any recent (within the past 6 weeks) bouts of dizziness, our findings highlight that anxiety-provoking situations that lead to enhanced conscious balance processing can induce distorted perceptions of instability in an older adult population. While dizziness is common in older adults [1], in at least 50% of cases, symptoms cannot be explained through traditional neurological or neuro-otological testing (and are, thus, ‘unexplained’) [6–8]. The present findings provide strong evidence for the role of attentional factors in the development of acute dizziness symptoms in this population. Future research should look to examine the relationship between fear of falling, conscious balance processing and persistent (unexplained) dizziness in older adults. Recent work has proposed associations between small vessel disease and unexplained dizziness [6]. As small vessel disease often leads to balance and gait impairments in older adults [33], perhaps these individuals develop a persistent fear of falling and consciously process their balance in an attempt to avoid a fall occurring; with such conscious processing influencing symptoms of dizziness. Future work should explore these possibilities.

While perceptions of stability significantly increased during Threat-Distraction compared to Threat (mean values of 78.1% vs. 70.2% stable, respectively), perceptions did not fully return to Baseline levels (mean of 90.6% stable). In contrast, conscious movement processing during Threat-Distraction (mean score of 10.6) fell below levels reported during both Baseline (mean of 11.5) and Threat (mean of 13.2). This implies that while conscious movement processing *can* influence perceived instability, this is not the sole mechanism underpinning (distorted) threat-related perceptions of instability. Previous research has reported increased sensory gain (specifically with regards to muscle spindle sensitivity [34, 35] and vestibular feedback [35–38]) when balance is threatened, which may result in anxious or fearful individuals perceiving themselves to be swaying at greater amplitudes than actually exhibited [39]. As self-reported levels of fear remained identical between Threat and Threat-Distraction, it is, therefore, likely that altered perceptions of sway resulting from increased sensory gain will persist—to some degree, at least—even when attention to balance is withdrawn. Alternatively, it is possible that fear and conscious movement processing influence different aspects of sensory processing (and, thus, uniquely contribute to perceptions of stability). For example, research has described how vestibular processing is altered during conscious balance processing (when induced independently from postural threat) in a manner that is seemingly different to that observed in participants fearful of falling [38, 40]. While it is difficult to isolate the extent to which conscious movement processing mediates the relationship between increased anxiety/fear and distorted perceptions of unsteadiness, the current work clearly identifies that such cognitive processes *can* mediate this relationship.

In the present work, increased postural threat was also characterised by a shortening of the transition window between open- and closed-loop postural control, indicating a lowered temporal threshold for sensory feedback during long-term closed-loop periods. We interpret these results to imply that individuals will display increased sensitivity to afferent feedback when they perceive their balance to be threatened. This may be related to increases in sensory gain (as described above) [35–37], given that such alterations would likely result in the central nervous system (CNS) having greater sensitivity for detecting small changes in body position during standing balance. It is worth also noting that shortened transition windows have been reported in individuals with functional dizziness [12, 13]. In this population, such postural responses are suggested to relate to increased conscious movement processing [12, 13]. However, our results suggest that smaller temporal transition windows between open- and closed-loop postural control may not be a consequence of increased conscious processing—at least, not in older adults during conditions of postural threat. On the contrary, a further shortening was observed when participants were prevented from consciously processing their balance (during Threat-Distraction). Collins et al. suggest that shorter transition periods between open- and closed-loop behaviour may reflect a more effective control mechanism allowing for earlier correction of drift in sway [30]. Thus, while greater conscious processing *may* contribute to the shortening of the transition period, it seems more likely that such behaviour reflects an automatic (and hypervigilant) mechanism triggered by the CNS in response to a postural threat.

As predicted, the postural threat manipulation also led to significantly greater high-frequency postural adjustments, compared to Baseline. Such observations are aligned with previous research exploring postural control in healthy young adults during conditions of heightened threat [22, 23, 25, 28, 39], as well as work conducted in individuals with functional dizziness disorders [11, 12]. We also observed significant increases in short-term diffusion coefficients during Threat. Combined, these behaviours likely reflect increased muscular co-contractions of the ankle muscles [13, 25, 28]. Recent work suggests that threat-related increases in high-frequency postural adjustments may be a consequence of greater conscious processing of balance [25]. By contrast, our results indicate that these behaviours may actually be underpinned by automatic postural control mechanisms. That is, we found that the increased sway frequency observed during Threat (compared to Baseline) coincided with significant increases in SampEn—a measure for which higher values reflect more ‘automatic’ (i.e. less consciously processed) postural control [29]. Moreover, when participants were prevented from consciously processing their balance (during Threat-Distraction), both sway frequency (especially Freq_high_) and SampEn further *increased*, while the critical time period showed further shortening (as described above). These changes were accompanied by substantial reductions in self-reported conscious processing; but were likely unaffected by changes in anxiety (as self-reported fear of falling and balance confidence remained unchanged). Collectively, these results suggest that postural threat results in enhanced automatic—and potentially adaptive—postural control mechanisms, a response that may be constrained by (excessive) attempts to consciously process balance. Although some degree of conscious processing may be necessary for older adults to maintain postural stability [41], future work should explore in greater detail the potential benefits and/or consequences of preventing conscious movement processing in this population during conditions of postural threat.

While the lack of clinical neurological or vestibular testing is a limitation of the present research, participants with diagnosed neurological and vestibular disorders were excluded, as were those who had experienced a recent bout of dizziness (within the past 6 weeks). As participants were asymptomatic (with respect to dizziness) at the time of participation, any undiagnosed or sub-clinical neurological/vestibular disorder is, therefore, unlikely to have confounded our results. Nonetheless, as we did not include a control group of older adults with dizziness caused by organic illness (e.g. benign paroxysmal positional vertigo), we are unable to determine the extent to which the observed results are specific to functional/unexplained dizziness or relevant for dizziness in general.

## Conclusion

The present work provides strong support for the assumption that increased conscious movement processing can underpin distorted perceptions of unsteadiness that occur when anxious or fearful of falling (and that also characterise functional dizziness disorders). Although fall-related anxiety/fear is a common trigger for conscious movement processing in older adults [16], other factors such as balance complaints, injury or age-related neurological changes (e.g. small vessel disease) may also lead to increased hypervigilance for processing aspects of balance in this population which may trigger or perpetuate feelings of dizziness [6]. Relatedly, in PPPD conscious processing of balance is likely triggered by an acute, episodic or chronic vestibular disorder (e.g. vestibular migraine), that may then be further exacerbated through increased anxiety [9–11]. Future work should confirm the degree to which conscious processing of balance predicts the severity of dizziness symptoms in both populations, and the extent to which symptoms are amendable through therapies that reduce such conscious processing.

## Data Availability

All analysed data are available via an Open Science Framework repository (https://osf.io/nvrky/?view_only=bcd27ccfe99c47feaff215173a76d2fc).

## Code availability

Custom MATLAB codes are available from the lead author following reasonable request.
